# Safety Early Warning Research for Highway Construction Based on Case-Based Reasoning and Variable Fuzzy Sets

**DOI:** 10.1155/2013/178954

**Published:** 2013-10-07

**Authors:** Yan Liu, Ting-Hua Yi, Zhen-Jun Xu

**Affiliations:** ^1^College of Architectural Engineering, Qingdao Agricultural University, Qingdao 266109, China; ^2^School of Civil Engineering, Dalian University of Technology, Dalian 116023, China

## Abstract

As a high-risk subindustry involved in construction projects, highway construction safety has experienced major developments in the past 20 years, mainly due to the lack of safe early warnings in Chinese construction projects. By combining the current state of early warning technology with the requirements of the State Administration of Work Safety and using case-based reasoning (CBR), this paper expounds on the concept and flow of highway construction safety early warnings based on CBR. The present study provides solutions to three key issues, index selection, accident cause association analysis, and warning degree forecasting implementation, through the use of association rule mining, support vector machine classifiers, and variable fuzzy qualitative and quantitative change criterion modes, which fully cover the needs of safe early warning systems. Using a detailed description of the principles and advantages of each method and by proving the methods' effectiveness and ability to act together in safe early warning applications, effective means and intelligent technology for a safe highway construction early warning system are established.

## 1. Introduction

China is currently one of the top infrastructure investors in the world. From zero highway breakthroughs in 1988 to the 74,100 kilometers of highway traffic mileage implemented by the end of 2010, comprising the second greatest highway network in the world; China has achieved a level of development that took western countries over 40 years to accomplish in only 22 years, realizing a historic breakthrough in highway construction. In keeping with an overall construction plan for an 850,000-kilometer highway road network [1], an increasing number of highway construction projects will come into operation over the next 10 years, the growth of which is unprecedented. While highways have generated significant economic benefits in the rapid development of the last 20 years, they have also resulted in billions of RMB of economic losses due to safety issues, highlighting the severe safety concerns in this industry.

**Figure 1:**
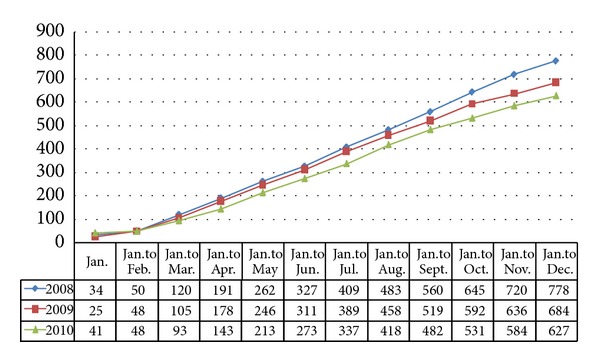
Number of safety accidents in the construction industry (2008–2010).

**Figure 2:**
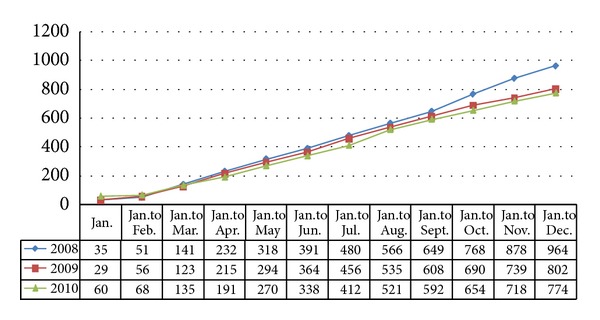
Death tolls from safety accidents in the construction industry (2008–2010).

According to the accident statistics for construction project safety issued by the Ministry of Construction shown in Figures 1 and 2, because China's related department strengthened management and improved managerial stuff educational level, the numbers of accidents and fatalities have been decreasing annually over the past three years. The total number of accidents and deaths is relatively large, and the number of people who have died of safety accidents in construction projects in China is 1.5 times that of the total death tolls in 50 other developed countries, including the United Sates, the United Kingdom, Germany, and Japan. The accident occurrences in road construction projects, which are a high-risk subindustry in construction, account for 34% of total construction project accidents, while the fatalities in this subindustry account for approximately 31% of all construction project fatalities and are caused by five types of accidents: height crashes, construction collapses, object attacks, electric shocks, and machinery injuries. The safety conditions in this subindustry are not satisfactory.

According to computations of static investments, it is estimated that the future capital required for national highway network construction is approximately 200 billion RMB. National highway construction will be occurring fairly rapidly until 2020. The annual investment was approximately 140 billion RMB until 2010 and will be approximately 100 billion RMB from 2010–2020. However, the direct and indirect losses caused by safety issues account for 2% of the annual total investment, which is a large figure that greatly hinders the development of road construction.

At first, the industry thought that the safety issues had purely incidental or unexplainable reasons, and concern for safety was limited to fatalities and property loss. With improved knowledge and concern for safety issues, the industry began to see that the occurrences were more or less related to incidents but also had their own laws and features. Because it has gotten a late start, the study of safety management in China is only an initial attempt in terms of both theory and practice, with imperfect on-site safety management materials, an indirect and hysteretic quality to safety effectiveness, and widespread uncertainties in construction projects. Thus, the importance of the construction safety work in China has been ignored for decades. So, there is an urgent need for current construction safety work to switch from accident handling after accidents to forecast at the initial stage, switch from handling the accident to predicting and preventing the accident, and switch from traditional management to modern scientific management. The key link to realizing this transition is construction safety early warning technology. The essence of safe early warning technology in construction projects lies in precontrol, prophase management, transitioning from accident handling to accident prevention, discovering and addressing potential risks at any time, and eliminating accidents in the early stages of a project. Therefore, early warning is one of the most effective methods of curbing accidents and reducing safety losses. In April 2011, Wang [2] noted at the 14th session of national construction safety officer working meetings that construction enterprises should establish and perfect safe production dynamic monitoring and early warning systems in addition to analyzing and auditing the hidden dangers and risks of their construction projects at regular intervals.

Further studies on early warning management models exist abroad and are focused mainly on macroeconomic pre-monitoring and microenterprise crises, such as an early warning study on financial crises [3, 4], computer network crises [5], and natural disasters, such as tsunamis and earthquakes [6, 7]. However, the study of industry production safe early warning, particularly early warning in construction projects [8], is relatively rare. A theoretical study of early warning in China must commence with the circular fluctuation of the economy in the middle of the 1980s [9] and then transition to the noneconomic early warning that has occurred in recent years, beginning with early warning management studies in the field of construction projects, such as coal mining [10, 11], bridge construction monitoring [12, 13], and deep excavation [14, 15]. Although the phrase “early warning” has been mentioned very frequently in other countries, systematic and in-depth studies are still rare and mostly focus on the computer technology involved in early warning management information systems.

The accident losses during highway construction in China over the past 20 years have been caused mostly by the lagging study and practice of safe early warning; thus, improving early warning abilities and preventing safety issues are now the industry's most challenging tasks.

## 2. Key Technology for Safe Early Warning Systems

Safety and risk are mutually contradictory and dependent in major construction projects. Safety risks do not exist alone on a microscale, and safety issues cannot be induced by a single risk element. In essence, safety is a systematic project containing subunits, such as safety risk forecasting, distinguishing safety risks, risk associations, risk element importance ranking, safety investment and effectiveness, safe early warning, safety evaluation, and an emergency response plan. On a macroscale, safety is related to construction progress, project quality, investment cost, and effectiveness, and these factors are interrelated and interact with one another, leading to an external action mechanism for safety issues.

Based on the current knowledge of safety, the selection of monitoring indices for corresponding early warnings should have a hierarchy. This paper divides early warning monitoring indices into a compulsory index hierarchy and dynamic index hierarchy. The compulsory index includes an average safety training time, safety education coverage rate, licensed personnel rate, site safety member rate, safety symbol installation rate, temporary electricity usage management standard rate, reasonableness of machinery material management, fire protection management standard rate, safety danger patrol, safe production meeting frequency, employment injury insurance coverage rate, height workload, and ecological conditions. The dynamic index includes deviations in the project progress and investment costs, soil stress changes and deformations, and variations in water level and environment. The improvement of safety awareness and safety standards is adopted for the compulsory indices in the early warning process, while such methods as reinforcing the monitoring of dangerous areas and time zones, qualitative and quantitative change monitoring of the index values, and the division of different warning districts are included in the dynamic indices. Once the analyzed data enter the warning districts, we can effectively curb safety issues with the different control measures that are taken according to the warnings made based on the level of severity. 

A complete and scientific safe early warning process includes the selection of monitoring indices and association analysis of the causes of the accident and warning degrees. Because a highway has such features as a one-off quality, uniqueness and a high level of uncertainty, the indices for early warning, accident association, and warning degree forecast should be uniquely based on the project features. Therefore, this paper introduces case-based reasoning (CBR) technology to the field of highway construction safe early warning systems to increase the accuracy and effectiveness of the technology. CBR is an important branch of artificial intelligence and originated in 1982 as part of Yale University Professor R. Schank's “Dynamic Memory," a book that created the basic theory of case-based reasoning. CBR is a similar or analogical type of reasoning that is designed to use existing experience and cases to solve new problems while also explaining the new situations. By accessing a knowledge base used to solve similar problems in the past, the current problem solutions are given an inference model or the use of old cases or experiences to solve new problems, evaluate new issues, explain atypical circumstances, or understand a new situation. CBR technology is used to solve a problem directly using previous examples of knowledge and can effectively solve difficult or problem areas that cannot be expressed otherwise. The self-learning function of CBR ensures the continuous enhancement of its reasoning, and it efficiently handles important items that are close or similar to the means [16]. However, papers involving both construction project safety and CBR are very rare—there are dozens abroad and less than 10 from China. However, these papers focus mainly on safety diagnosis, quality control, and slope stabilization and accident emergency response, and none are deep or thorough enough. Based on the advantages of CBR in project applications and its high accountability and communicability, a 2010 key scientific project regarding major accident prevention and solution technologies for safe production, issued by China's national safety supervision bureau, discusses CBR and shows us that CBR technology is increasingly used in construction safety studies. 

**Figure 3:**
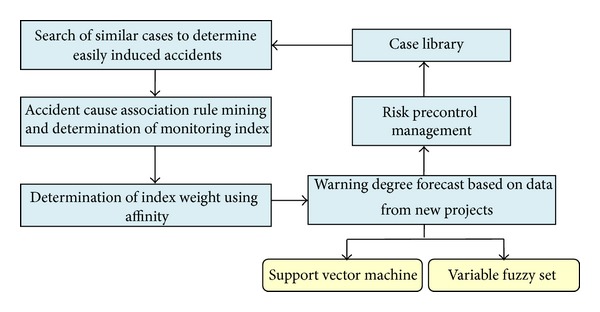
CBR-based highway construction safe early warning system.

Timely and accurate early warning systems can effectively reduce the occurrence of accidents and eliminate safety losses while maximizing the effectiveness of safety investments. This study is based on case-based reasoning technology and researches three key links of early warning systems, as shown in Figure 3, which is a virtuous cycle process of self-learning. Using the analogical reasoning-based features of CBR, the key to the application is in searching former cases that are similar to new projects because experiences from previous similar cases are more thorough and accountable and more severe or potential risks can be mined and identified. Therefore, we should search existing cases with similar control properties to the new projects, in which control properties can be set as indices, such as project type, construction technology, geological conditions, and methods of precipitation and water drainage. Next, we can calculate the similarity of comparative properties, such as construction costs and project kilometers, based on the search, filtering finished projects for which the similarities surpass a minimum threshold. The risks and accidents experienced by the similar projects can be summarized and used as keywords to search a case library, mining risk associations that lead to accidents, and then strongly correlated associations exceeding the minimum threshold of association rules' support and confidence as an accident-prone frequent item can be set to reinforce monitoring. This study uses the association degree to determine index weight. The greater the relationship to risk accidents is the heavier the index weight becomes. Because the indices have different types and associations, this paper uses a support vector machine with a strong generalization capacity and variable fuzzy set approach to perform the warning degree forecast and assure the accuracy of the warnings. These two methods have excellent theoretical superiority and comparatively lagging applications, so this paper combines cases to analyze and verify application effectiveness based on the two methods' principles and advantages.

## 3. Association Rules

Association rule mining is one of the most active directions of study in data mining, which is an important Knowledge Discovery in Database (KDD) research subject initially proposed by Ramakrishnan et al. [17]. Data mining reflects interesting or relevant associations among projects from a large database. With the increasing scale of data collected and stored in data libraries, people are becoming more interested in the mining of relevant association knowledge from these data.

There are two important concepts in the algorithm of association rule, support and confidence. If the proportion of objects *A* and *B* in data library *D* is *s*, then we can say that the support of the association rule for *A* and *B* in *D* is *s*, support(*A* → *B*) = support(*A* ∪ *B*) = *P*(*A*, *B*). If the proportion of data library *D* containing objects *A* and *B* at the same time is *c*, then we can say that the confidence of the association rule for *A* and *B* is *c*, confidence(*A* → *B*) =    support(*A* ∪ *B*)/support(*A*) × 100%, or *P*(*B*|*A*). The support reflects the importance of association rules in the data library, and the confidence measures the accountability of the association rule. 

Using association analyses from previous construction projects, Chen [18] applied the grey association analysis approach to distinguish between the association elements affecting safety preevaluation systems and sequence the primary and secondary associated danger levels of dangerous substances, thus solving the uncertainty and accountability issues in safety accidents. Sawacha et al. [19] analyzed numerous accident samples and summarized the top 5 important elements associated with on-site safe production. Siu and his colleagues [20] made a comparative analysis of their associations from personal elements and accident rates, while Halperin and McCann [21] determined relevant elements from the study of frequent accident locations. Case-based reasoning association rules are different from the association analysis performed in the literature because references provided by similar cases can more accurately reflect the dependence and association between a monitoring index and risk events. In the mining process for early warning rules, we first set the minimum threshold for the support and confidence of the association rules. Then, we search all of the high-frequency risk sets related to safety issues in the case library and generate strongly correlated rules from these cases. 

This study uses relational algebra theory-based association rules to perform risk association mining, and the algorithm only needs to scan the data library once (overcoming the classic Apriori algorithm's weakness of needing to scan a data library multiple times) and has good concurrency and scalability. Assuming that *D* is the case library and *T* = {*t*_1_, *t*_2_, *t*_3_,…, *t*_*m*_} and *I* = {*i*_1_, *i*_2_, *i*_3_,…, *i*_*n*_} are the case set and risk itemset, respectively, the matrix is as follows:

(1) $$\begin{array}{c} R=\left[\begin{array}{c} r_{11},r_{12},\ldots ,r_{1n}\;\\ r_{21},r_{22},\ldots ,r_{2n}\\ \vdots \\ r_{m1},r_{m2},\ldots ,r_{mn} \end{array}\right], \end{array}$$

which stands for the binary relation from *T* to *I*.

In the formula, the value of *r*_*ij*_ (*i* = 1,2,…, *m*; *j* = 1,2,…, *n*) is 1 or 0, representing whether case *i* includes risk element *j*. ∑_*i*=1_^*m*^ *r*_*ij*_/*m* is the support of property *j* for the 1st set. If the support is bigger than the minimum threshold, then the risk item element is 1 large itemset. If an itemset is not large, then any sets including this itemset can never be large. Therefore, 2 large itemsets must search based on 1 large itemset. Assume that *A* is a 1 large itemset, *a*_*i*_ stores 1 large relevant itemset, and *i* = 1,2,…, *s*, so *A* has *s* elements. ∑_*p*=1_^*m*^(*r*_*pa*_*i*__ and *r*_*pa*_*j*__)/*m* is the support of 2 itemsets {*a*_*i*_, *a*_*j*_}, so the support must be larger than the minimum threshold to be 2 large itemsets. These conditions apply to all itemsets. If there exists an item *V* to make ∑_*p*=1_^*m*^(*r*_*pi*_*k*−1__ and *r*_*pv*_)/*m* larger than the minimum support threshold, then {*i*_1_, *i*_2_,…, *i*_*k*−1_, *ν*} is a *k* large itemset.

This paper considers height crash accidents, which have the highest occurrence and number of fatalities, as an example.  Table 1 represents cases similar to the 12 height crash accidents obtained from the case library and their risk associations. *A*–*E* represent separate risk elements, such as safety belt failure or lack of safety belt use, strut damage, loss of body control, safety facility failure, and safety net damage.

The algorithm is described in MATLAB R2007a as in Algorithm 1.

Set the minimum support threshold *t* of this early warning association rule to 40%. Then, the 1 large itemset from this algorithm is {*A*}, {*B*}, {*C*}, {*D*}, and {*E*}; the 2 large itemset is {*A*, *B*}, {*A*, *C*}, {*A*, *D*}, {*A*, *E*}, {*B*, *C*}, {*B*, *D*}, {*B*, *E*}, {*C*, *D*}, {*C*, *E*}, and {*D*, *E*}; and the *k* large itemset is {*A*, *B*, *C*}, {*A*, *B*, *E*}, {*A*, *C*, *E*}, {*B*, *C*, *D*}, {*B*, *C*, *E*}, and {*A*, *B*, *C*, *E*}, which is the same as the results gained from the classic Apriori algorithm. A *k* large itemset can typically represent the mechanism of action, so curbing the occurrence of a *k* large itemset is key to safe early warnings. The rules set by this association algorithm are fixed, so if we can use it as a base and combine quantitative data, such as the probability of basic events and accidents, sensibility of basic events, safety thresholds, or safety investment effectiveness, the risk element association rules can be further deduced.

## 4. Support Vector Machine

The accuracy of a warning degree forecast decides the pertinence of safety precontrol measures and the effectiveness of safety investments. Different warning degrees indicate different measures and investment costs. Therefore, safe early warning systems have strict classification method requirements to make full use of investment costs, effectively control risks, and avoid accidents. The interpretation of a neural network does not give it the ability to learn and can easily cause weak generalization characteristics. To combat this tendency, this study introduces the most successful statistical learning theory, support vector machine technology. The support vector machine (SVM) solves small samples with nonlinear and high-dimensional pattern recognition performance, giving it many unique advantages. Cortes and Vapnik [22] first proposed the SVM in 1995 and based it on statistical learning theory, and the theory of VC dimension is based on the structural risk minimization principle according to the limited sample information in the model complexity (the learning accuracy of a particular training sample, or accuracy) and the learning ability (error-free samples that identify any capacity) to establish the best compromise between the two and obtain the best generalization capability (or generalization) [23]. The main advantages of SVM technology are that its small samples can solve machine learning problems, improve generalization performance, solve high-dimensional problems and nonlinear problems, and avoid neural network structure selection and local minimum problems.

Experiments have shown that the results of fitting a low-order function are better than the results of fitting a higher-order function in noisy conditions, even if the true model occurs several times [24]. Thus, attempting to use a very complicated model to fit a limited sample, even with the “optimal" function, results in the loss of generalization ability in low-dimensional space.

**Figure 4:**
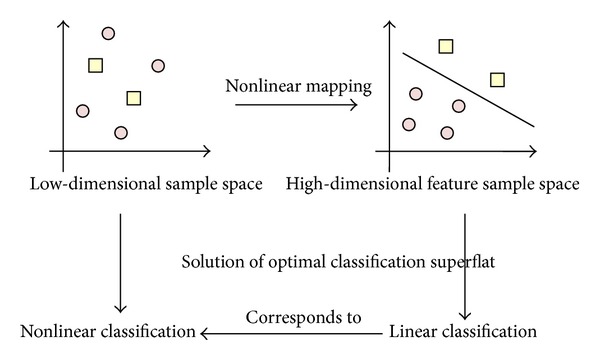
Principles of support vector machine thinking.

Unlike traditional statistical methods, the SVM defines structural risk minimization as its goal and makes a good pre-selection using a nonlinear transformation, nuclear function, and low-dimensional input vectors mapped into a high-dimensional feature space. An optimal separating hyper plane can be constructed in this feature space. In other words, the promotion of a high-dimensional space constructed with a low-dimensional space produces more powerful functions, as shown in Figure 4.

**Figure 5:**
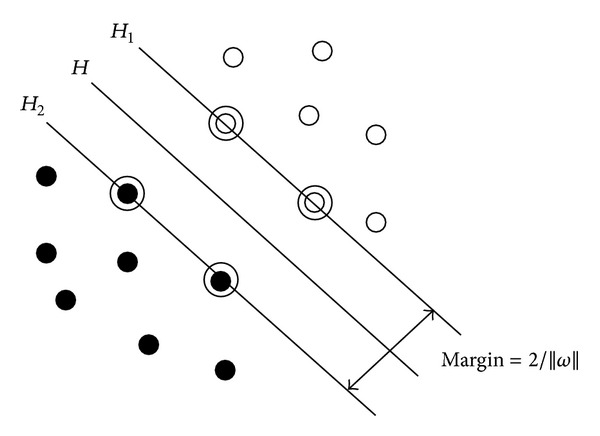
Linearly separable cases with optimal classification lines.

The SVM two-dimensional realization of the situation in Figure 5 can be used to explain its use. The solid and hollow points represent two samples *H* for the classification line, *H*_1_ and *H*_2_, respectively, from the classification of various line types in a sample of recent data. In the classification of lines parallel to the straight line, the distance between the lines is called the classification interval (margin). The so-called optimal separating line requires that the correct classification of a line not only be capable of separating the two line types (a training error rate of 0) but also be capable of classifying the largest interval, or the promotion of capacity control, which is one of the core concepts of the SVM.

The classification line for the equation of *H* is *x* · *ω* + *b* = 0, where *H*_1_ and *H*_2_ are classes 1 and −1, respectively, and the equations of *H*_1_ and *H*_2_ are *x* · *ω* + *b* = *y*, *y* = 1 and *x* · *ω* + *b* = *y*, respectively, with *y* = −1. The determination of whether the sample belongs to class 1 or class −1 can be summarized as

(2) $$\begin{array}{c} y_{i}\left[\left(\omega \cdot x_{i}\right)+b\right]-1\geq 0,\;i=1,2,\ldots ,n. \end{array}$$

The interval classification is equal to 2/||*ω*||, so the maximum interval is equivalent to the minimum ||*ω*||^2^. Therefore, (2) the constraints to meet the minimum are ||*ω*||^2^/2, the classification of surface is called the optimal separating surface, and *H*_1_ and *H*_2_ point to the training samples, called support vectors.

Because the presence of noise will not distinguish between some samples, even if the low-dimensional vector is mapped to a high-dimensional feature space, the introduction of slack variables *ζ*_*i*_ and a penalty factor *C* represent that the data noise in the fault tolerance of the SVM achieves better classification results. The purpose of the representation is to allow part of the introduction of the point that does not meet the requirement that the outliers give up. The resulting generalized optimal separating line model is

(3)    min⁡    ||ω||22+C∑i=1lζi subject  to   yi[(ω·xi)+b]1−ζi (i=1,2,…,l)     (where  l  is  the  number  of  samples) ζi≥0.

This equation can be transformed into a dual problem for its resolution, and because it is a convex quadratic programming problem, there exists a global optimal solution.

In summary, the SVM training error and generalization, according to the limited sample information in the model complexity, find the best compromise to solve for small samples in nonlinear, high-dimensional problems, such as pattern recognition. Although the SVM is widely used and the method has many unique advantages, research into its use is still relatively lagging. This paper introduces the use of the SVM method into case-based reasoning for construction safety warning degree forecasts, preserving the objectivity of actual risk elements while maintaining the forecast accuracy of warning degrees, achieving target precontrol measures, and avoiding accidents.

This paper takes the historical data from [25] (Table 2) as an example and considers a case study of vector machine applications in safe early warning systems.

The process can be described in MATLAB R2007a as follows:

 %% Support Vector Machines
 [bestacc, bestc, bestg] = SVMcgForClass (train_lable, train, cmin, cmax, gmin, gmax,…, v, cstep, gstep, accsetp)
 % cross-validation to determine the optimal penalty factor *c* and nuclear function parameter *g* model = svmtrain (train_label, train, [“libsvm_options”])
 % determine the training set classification
 [predict, acc] = svmpredict (test_label, test, model)
 % by category model to classify new samples

**Figure 6:**
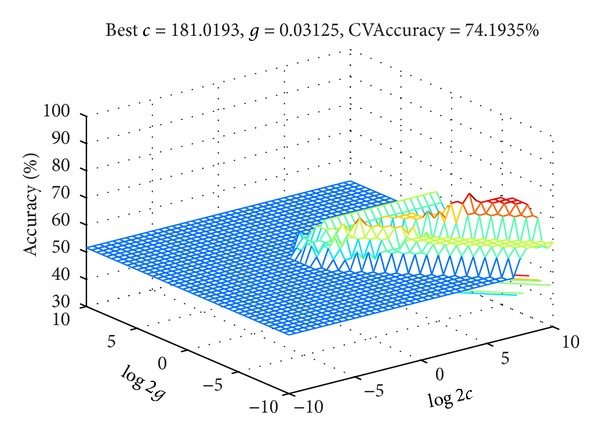
Cross-validation optimized three-dimensional display.

This case makes the warning degree its object. Levels 1–3 represent slight, moderate, and severe warnings, respectively, and the other 7 elements represent the risk properties, forecasting the warning degree of this case through the support vector machine classifier. The first 31 cases are set as training samples, and the final 5 are set as testing samples. In the classification setting, the kernel function selects a radial basis function while optimizing the parameters of the cross-validation process. The training samples are randomly divided into 5 groups, and for the maximum number that appears, the cross-validation accuracy of the smallest group of *c* is selected because the high penalty parameter causes the algorithm to learn and is not conducive to the generalization of the results. Based on this result, the best penalty parameter *c* = 181.0193 and the best RBF kernel parameter *g* = 0.03125, for which the highest cross-validation accuracy rate is 74.1935%, are shown in Figure 6.

In the final training set, the forecast results show that the classification accuracy of the classifiers trained by this set is 90.3226%, and the testing set samples have an accuracy of 100%, with the entire classification process lasting only 3.96 seconds. At the same time, the accuracy of the BP neutral network approach for this testing set is only 60%, and it takes 22.48 seconds to finish the classification, which indicates the generalization capacity strength of the SVM. The SVM has major advantages over neutral networks in terms of its forecast accuracy and efficiency and can efficiently improve the pertinence of precontrol measures and the effectiveness of safety investments.

## 5. Variable Fuzzy Qualitative Change Criterion Mode

With the existence of a dynamic index, safe early warnings must be a process of dynamic monitoring. As the project progresses, the index values will change dynamically among warning districts, with some changing across warning districts and some changing only within warning districts. Therefore, the index values are a critical test for illustrating warning accuracy and disguising whether the index change is a quantitative or qualitative change. Professor Chen proposed relative difference function-based variable fuzzy sets [26–28] with quantitative and qualitative change (i.e., gradual and abrupt) criterion modes [29].

**Figure 7:**
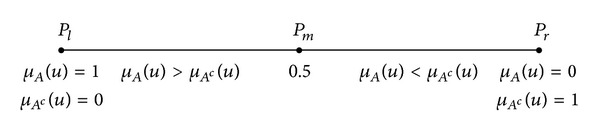
Opposite fuzzy set schematic.

Assume that for any element *u* (*u* ∈ *U*), there is a vague concept *A* in the universe of discourse *U* at any point on the reference continuum axis of the relative membership function. The relative membership of *U* to *A* is *μ*_*A*_(*u*) and *μ*_*A*^*c*^_(*u*) to *A* ^*c*^, the opposite concept of *A*, and *μ*_*A*_(*u*) + *μ*_*A*^*c*^_(*u*) = 1. Among these variables, 0 ≤ *μ*_*A*_(*u*) ≤ 1 and 0 ≤ *μ*_*A*^*c*^_(*u*) ≤ 1. As shown in Figure 7, the left pole *P*_*l*_ has *μ*_*A*_(*u*) = 1 and *μ*_*A*^*c*^_(*u*) = 0, the right pole *P*_*r*_ has *μ*_*A*_(*u*) = 0 and *μ*_*A*^*c*^_(*u*) = 1, and *P*_*m*_ is the gradual qualitative change in the point of reference continuum interval [1,0] (for *A*) and [0,1] (for *A* ^*c*^), meaning that *μ*_*A*_(*u*) = *μ*_*A*^*c*^_(*u*) = 0.5.

**Figure 8:**
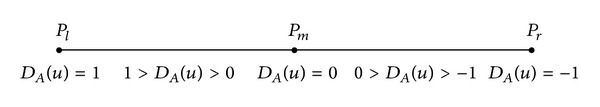
Relative difference function schematic.

Assume that *D*_*A*_(*u*) = *μ*_*A*_(*u*) − *μ*_*A*^*c*^_(*u*) is called the relative difference of *U* to *A*. As shown in Figure 8:

 If *μ*_*A*_(*u*) > *μ*_*A*^*c*^_(*u*), then 1 > *D*_*A*_(*u*) > 0;
 If *μ*_*A*_(*u*) = *μ*_*A*^*c*^_(*u*), then *D*_*A*_(*u*) = 0;
 If *μ*_*A*_(*u*) < *μ*_*A*^*c*^_(*u*), then −1 < *D*_*A*_(*u*) < 0.

Assume that $\underline{V}=\{(u,D)|u\in U,D_{A}(u)=\mu_{A}(u)-\mu_{A^{c}}(u),D\in [-1,1]\}$.

$\underline{V}$ is called the fuzzy variable set and *A*_+_, *A*_−_, and *A*_0_ are called the attraction basin (main), rejection basin (main), and gradual qualitative change boundary, respectively.

Assume that *C* is the variable element set of $\underline{V}$ and *C* = {*C*_*A*_, *C*_*B*_, *C*_*C*_}, where *C*_*A*_  is a variable model set, *C*_*B*_ is a variable model parameter set, and *C*_*C*_ is the other variable element set excluding the model and its parameters.

When summarizing the above statement, we can conclude that the criterion modes of the variable fuzzy qualitative and quantitative changes are as follows.

1. If *D*_*A*_(*u*) > 0 and *D*_*A*_(*C*(*u*)) > 0, then *D*_*A*_(*u*) · *D*_*A*_(*C*(*u*)) > 0 is a quantitative change.
2. If *D*_*A*_(*u*) > 0 and *D*_*A*_(*C*(*u*)) < 0, then *D*_*A*_(*u*) · *D*_*A*_(*C*(*u*)) < 0 is a gradual qualitative change (through *D*_*A*_(*u*) = 0).
3. If *D*_*A*_(*u*) < 0 and *D*_*A*_(*C*(*u*)) < 0, then *D*_*A*_(*u*) · *D*_*A*_(*C*(*u*)) > 0 is a qualitative change.
4. If *D*_*A*_(*u*) < 0 and *D*_*A*_(*C*(*u*)) > 0, then *D*_*A*_(*u*) · *D*_*A*_(*C*(*u*)) < 0 is a gradual qualitative change (through *D*_*A*_(*u*) = 0).

Therefore, we can conclude that if *D*_*A*_(*u*) > 0 or *D*_*A*_(*u*) < 0, the criterion modes of the quantitative and gradual qualitative changes of the variable fuzzy set are *D*_*A*_(*u*) · *D*_*A*_(*C*(*u*)) > 0 and *D*_*A*_(*u*) · *D*_*A*_(*C*(*u*)) < 0, respectively.

As shown in Figure 8, the left and right poles *P*_*l*_ and *P*_*r*_, where *D*_*A*_(*u*) = 1 and *D*_*A*_(*u*) = −1, are both abrupt qualitative change boundaries. If *D*_*A*_(*u*) > 0, *D*_*A*_(*u*) changes from a certain positive value to “1” (abrupt change), then *D*_*A*_(*u*) · *D*_*A*_(*C*(*u*)) = |*D*_*A*_(*u*)| is an abrupt qualitative change (without *D*_*A*_(*u*) = 0). If *D*_*A*_(*u*) < 0, *D*_*A*_(*u*) changes from a certain negative value to “−1” (abrupt change), then −|*D*_*A*_(*u*)| · *D*_*A*_(*C*(*u*)) = |*D*_*A*_(*u*)| is also an abrupt qualitative change (without *D*_*A*_(*u*) = 0).

Therefore, we can see that the criterion mode for abrupt qualitative change without *D*_*A*_(*u*) = 0 can be summarized as *D*_*A*_(*u*) · *D*_*A*_(*C*(*u*)) = |*D*_*A*_(*u*)|.

As this process continues, the criterion mode for abrupt qualitative change with *D*_*A*_(*u*) = 0 can be summarized as *D*_*A*_(*u*) · *D*_*A*_(*C*(*u*)) = −|*D*_*A*_(*u*)|.

Because current construction projects tend to excessively favor internal indices in dynamic index monitoring for safe early warning systems, the abnormal state of external indices, such as cost and progress, can also have a negative effect on safety situations. This study uses the changes in construction progress and investment for the 210 road section of a national highway as an example to verify the effectiveness of this qualitative and quantitative change model [30]. This road starts at the Qiujiahe River on the cross-boundary between Sichuan and Chongqing and ends at Heishizi in the Jiangbei District of Chongqing, connecting with the Yuchang highway. The highway has a total length of 53.108 kilometer. Tables 3 and 4 summarize the progress and investment costs, respectively, for Contract F of this project.

The linear formula for the relative difference in this application document [31] is

(4) $$\begin{array}{c} D_{A}\left(u\right)=\left(\frac{x_{i}-b_{i}}{a_{i}-b_{i}}\right)\;x_{i}\in \left[a_{i},b_{i}\right],\\ D_{A}\left(u\right)=\left(\frac{x_{i}-b_{i}}{d_{i}-b_{i}}\right)\;x_{i}\in \left[b_{i},d_{i}\right], \end{array}$$

where *x*_1_ is the investment cost deviation rate, *x*_2_ is the progress deviation rate, the comprehensive evaluation of risk is based on the two deviation rates, and the relative differences between the investment cost and progress from February 2003 to September 2003 are calculated separately according to the [*a*, *b*] and [*b*, *d*] interval eigenvalues in Table 5.

Assume that the weight vector of the two indices is *ω* = (0.5,0.5). Then, the relative difference in monthly risk is *D*_*A*_(*u*) = ∑_*i*=1_^2^ *ω*_*i*_ · *D*_*A*_(*u*)_*i*_.  Table 6 shows the monthly comprehensive relative difference. 

The closer the value of *D*_*A*_(*u*) is to −1, the greater the risk and the greater the pressure for safety are. The closer the value is to 1, the safer the project is. Based on the results in Table 6, we can see the change in tendency to risk. The safety level experiences a gradual qualitative change from April to May, while the changes from February to April and from May to September are both quantitative. The change is negative from February to April, indicating that the deviation in progress and investment costs during this period have a negative effect on safety production, and the change from May to September is positive, indicating that the deviation in progress and investment costs during this period does not have a negative external effect on safety situations. The results are simple and intuitive, and the existence of association rules means that the occurrence of safety issues is a combined action of multiple risk elements. Therefore, it is far from sufficient to set a warning degree threshold for individual risk elements to ensure safety when monitoring dynamic indices. This method can also be applied to the dynamic monitoring of multiple indices and intervals. We can create corresponding control measures based on the results, thereby curbing safety issues. 

## 6. Conclusions

Early warning technologies are used to determine both safety situations and safety losses. Good early warning technologies can not only reduce losses by limiting the available accident sources but can also indirectly lower investment costs by guiding safety input benefit maximization. Different from other existing research, the following conclusions and recommendations are made based on this research.

1. By using analogical reasoning-based CBR, this paper gives a basic schematic for solutions to safe early warning technologies by practically solving the three-key issues of index selection, accident cause association analysis, and warning degree forecast, which also penetrate the whole process of safe management.
2. Combined with the characteristics of highway projects, as well as the possible problems in the process of data processing, this paper introduces association rule mining, support vector machine classifiers and variable fuzzy qualitative and quantitative change criterion modes in order to keep the data of high fidelity. Together with experiments proving the effectiveness of the methods, the proposed method is a completely feasible and effective means of improving our country's early warning technologies.
3. With the gradual application of artificial intelligence to the security of construction projects, the CBR technology can be applied to safe early warning systems for construction projects in our country. However, research shows that a lack of existing cases and the complexity of the data are the biggest bottlenecks in the application of CBR. Therefore, further study of CBR technology and the settlement of data processing, case statistics, and searches of highway construction safe early warning systems are key to improving the practicability of safe early warning systems and safety management.

## Figures and Tables

**Algorithm 1:**
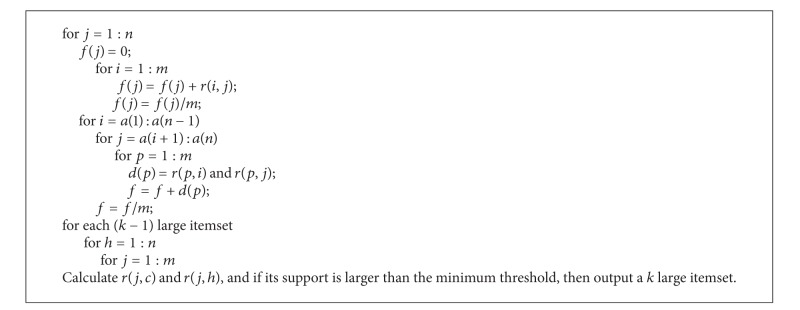


**Table 1:** Risk associations for height crash accidents.

*T*ID	Items	*A*	*B*	*C*	*D*	*E*
*T*1	{*A*, *B*, *C*, *D*, *E*}	1	1	1	1	1
*T*2	{*A*, *B*, *C*, *D*}	1	1	1	1	0
*T*3	{*A*, *B*, *C*, *E*}	1	1	1	0	1
*T*4	{*A*, *B*, *D*}	1	1	0	1	0
*T*5	{*A*, *B*, *C*, *D*, *E*}	1	1	1	1	1
*T*6	{*A*, *B*, *C*, *E*}	1	1	1	0	1
*T*7	{*A*, *D*, *E*}	1	0	0	1	1
*T*8	{*B*, *D*}	0	1	0	1	0
*T*9	{*A*, *B*, *C*, *E*}	1	1	1	0	1
*T*10	{*B*, *C*, *D*, *E*}	0	1	1	1	1
*T*11	{*A*, *C*, *D*, *E*}	1	0	1	1	1
*T*12	{*B*, *C*, *D*}	0	1	1	1	0

**Table 2:** Historical data sheet.

Project number	Warning degree	Workload when working at height	Safety protection level	Engineering geological conditions	Installation and usage of machinery	Workers without safety training	Organizational ability	Safety material status
1	2	0.3	82	2	82	0.14	68	1
2	2	0.38	64	0	72	0.15	84	1
3	1	0.2	73	2	93	0.11	84	1
4	1	0.34	83	2	91	0.12	82	2
5	2	0.22	74	0	94	0.1	88	2
6	1	0.32	94	2	95	0.2	80	2
⋮	⋮	⋮	⋮	⋮	⋮	⋮	⋮	⋮
30	1	0.34	85	2	83	0.12	79	0
31	2	0.35	65	2	81	0.08	70	2
32	1	0.3	90	1	85	0.15	88	2
33	2	0.35	72	1	72	0.14	65	1
34	1	0.22	68	1	85	0.04	88	1
35	1	0.25	85	2	80	0.12	85	1
36	2	0.35	70	1	68	0.14	70	1

**Table 3:** Basic information on Contract F (2003.02–2003.09) 10,000 Yuan.

Time	Month	Plan for this month	Actual progress during this month	Total Progress since commencement	Percentage of actual progress to planned progress	Change in contract price
2003.2	14	880.1126	597.9812	5991.5486	0.68	8695.2054
2003.3	15	938.522	717.9559	6709.5045	0.765	8555.2054
2003.4	16	918.4406	530.2455	7239.75	0.58	8555.2054
2003.5	17	959.0213	630.8527	7820.6027	0.6578	8655.2054
2003.6	18	766.6541	218.3175	8029.8021	0.2848	9021.8495
2003.7	19	766.6541	259.7389	8219	0.3388	9221.8495
2003.8	20	68.6561	391.8619	8611.4029	0.586	9221.8495
2003.9	21	593.301	182.7692	8774.9369	0.308	9141.8495

**Table 4:** Investment cost risk calculations for Contract F 10,000 Yuan.

Time	Number of project changes this month	Investment cost deviation value (accumulated Change)	Earned value	Investment cost deviation rate	Progress deviation rate
2003.2		−1,614.1201	7,605.669	−0.2122	−0.1856
2003.3	−140	−1,754.1201	8,463.625	−0.2073	−0.2050
2003.4	0	−1,754.1201	8,993.87	−0.1950	−0.2050
2003.5	100	−1,654.1201	9,474.723	−0.1746	−0.1911
2003.6	366.6441	−1,287.476	9,317.278	−0.1382	−0.1427
2003.7	200	−1,087.476	9,306.476	−0.1169	−0.1179
2003.8	0	−1,087.476	9,698.879	−0.1121	−0.1179
2003.9	−80	−1,167.476	9,942.413	−0.1174	−0.1277

**Table 5:** [a, b] and [b, d] interval eigenvalues for the deviation index.

x_1_		x_2_	
[a_1_, b_1_]	[b_1_, d_1_]	[a_2_, b_2_]	[b_2_, d_2_]
[−0.1, −0.18]	[−0.18, −0.25]	[−0.1, −0.18]	[−0.18, −0.25]

**Table 6:** Relative differences for Contract F (2003.2–2003.9).

Time	2003.2	2003.3	2003.4	2003.5	2003.6	2003.7	2003.8	2003.9
*D*_*A*_(*u*)	−0.27	−0.37	−0.27	0.26	0.26	0.78	0.80	0.76
